# Detection of Anterior Hyaloid Membrane Detachment Using Deep-Range Anterior Segment Optical Coherence Tomography

**DOI:** 10.3390/jcm11113057

**Published:** 2022-05-28

**Authors:** Haruhiro Mori, Yuta Ueno, Shinichi Fukuda, Tetsuro Oshika

**Affiliations:** Department of Ophthalmology, Faculty of Medicine, University of Tsukuba, Tsukuba 305-8577, Ibaraki, Japan; moririn21@gmail.com (H.M.); yu_ueno71@yahoo.co.jp (Y.U.); caesar.shihtzu@gmail.com (S.F.)

**Keywords:** anterior hyaloid membrane, Berger’s space, anterior vitreous detachment, anterior segment optical coherence tomography

## Abstract

The visibility of anterior hyaloid membrane (AHM) and Berger’s space in phakic eyes was investigated. In 624 eyes of 624 patients, the retrolental space was scanned with the deep-range anterior segment optical coherence tomography (AS-OCT, CASIA2, Tomey). Subgroup analysis was conducted in 223 eyes undergoing cataract surgery. The logistic regression analysis using the backward-elimination method was performed to evaluate the influence of various factors on the visibility of AHM (dependent variable). Explanatory variables included age, gender, axial length, corneal power, corneal cylinder, and the Co III gradings. Intrarater repeatability for AHM visibility was excellent with the prevalence-adjusted bias-adjusted kappa (κ coefficient) of 0.90. AHM was observed in 43 eyes (6.9%). The patients with visible AHM (68.1 ± 8.8 years) were significantly older than those without visible AHM (52.6 ± 25.6 years) (*p* < 0.001). The logistic regression analysis in the cataract surgery subgroup revealed that axial length (*p* = 0.030) and corneal power (*p* = 0.043) were significantly associated with AHM visibility. The mean absolute refractive error from target was significantly larger in eyes with visible AHM (0.670 ± 0.384 D) than with invisible AHM (0.494 ± 0.412 D) (*p* = 0.037). The postoperative refractive prediction was less accurate in eyes with visible AHM, but no significant tendency existed in terms of myopic or hyperopic shifts.

## 1. Introduction

The anterior surface of the vitreous is known as the anterior hyaloid membrane (AHM), which is a thin-layered structure that runs from the pars plana and firmly adheres to the posterior lens capsule by means of the hyaloideocapsular ligament of Wieger [1,2]. Berger’s space lies at the center of the anterior vitreous within the hyaloideocapsular ligament. In most adults, this is a potential rather than a true space [3], with the posterior capsule and anterior hyaloid face directly apposed in normal eyes. When there is fluid in Berger’s space, it is thought to be due to an age-related or pathological degeneration of the ligament of Wieger that connects the space with Petit’s canal, allowing aqueous to enter [4,5].

Since vitreolenticular adhesion is considered to stabilize the posterior lens capsule and also prevent the irrigation fluid to flow into the Barger’s space during cataract surgery, AHM detachment can displace the posterior capsule anteriorly, elevating the risk of intraoperative complications [6]. In contrast to the posterior hyaloid membrane that can be easily visualized with optical coherence tomography (OCT), however, AHM and Berger’s space in normal human eyes are rarely seen in vivo. Several attempts have been reported to visualize AHM during surgery using a fiberoptic endoscope [7] and intraoperative OCT attached to surgical microscope [4,6]. Berger’s space was visualized in eyes with pathological conditions such as trauma and pigment dispersion syndrome [8,9,10,11], or after the capsulo-hyaloidal hydroseparation procedure with the aid of triamcinolone enhancement [12]. Apart from these limited circumstances, little is known about the physiological status of AHM and Berger’s space [13].

Recently, deep-range anterior-segment OCT (AS-OCT) was developed that has expanded the vertical imaging depth to 13 mm, compared to 6 mm depth with the conventional AS-OCT. This new generation of AS-OCT enables in vivo imaging of the whole crystalline lens in its full length including the posterior lens capsule, and its research applications include measurements of crystalline lens thickness and radius of curvature of the posterior lens surface [14,15,16]. We conducted the current study to assess the visibility of AHM and Berger’s space using deep-range AS-OCT in normal human eyes.

## 2. Materials and Methods

### 2.1. Patients

We retrospectively collected the data of patients who had undergone ocular examinations at Tsukuba University Hospital from July 2018 to August 2020. All eyes were phakic and underwent AS-OCT imaging. Eyes were excluded from the study if they had corneal opacity, any history of eye surgery including cataract surgery, or past history of ocular trauma. Eyes with advanced cataract that hinders AS-OCT measurements through the crystalline lens were also excluded.

Subgroup analysis was conducted in eyes that were undergoing cataract surgery. In this group, eyes with any apparent surgical risks were excluded, such as exfoliation syndrome, phacodonesis, pupil diameter <6.0 mm after pharmacological mydriasis, and α_1_ adrenergic blocker medication for benign prostate hypertrophy.

The Institutional Review Board of the Tsukuba University Hospital waived the need for IRB approval due to the retrospective nature of the study. Nevertheless, its approval was obtained for the retrospective review of the patients’ clinical record. This study was conducted in accordance with the Declaration of Helsinki.

### 2.2. Evaluation of Anterior Hyaloid Membrane

The retrolental space was examined using CASIA2 (Tomey, Nagoya, Japan), a swept-source AS-OCT system, which provides 50,000 A-scans per second and takes only 0.018 s to capture a single cross-sectional image (800 A-scans per cross-sectional image). Tomographic images of the lenses were obtained by using the lens biometry mode. In this mode, the scan range is 16 mm × 13 mm (transverse × depth), and 16 images are taken in one assessment.

AS-OCT imaging was performed after pharmacologic pupillary dilatation. The participants were instructed to focus on an internal fixation light during the scanning. After adjusting the patients’ position, eyes were scanned in two-dimensional mode with the auto-alignment function. The examiners viewed the scans to ensure the quality of the images. One experienced reader (H.M.) scrutinized the obtained images and judged the visibility of a membranous structure apart from the posterior lens capsule as described in a previous histopathological study [7]. The reader was allowed to adjust the brightness and contrast of images on the screen display to maximize the visibility of intraocular structures.

Intrarater reliability was assessed by calculating the prevalence-adjusted, bias-adjusted kappa (κ coefficient) (PABAK) [17] in 200 eyes, in which visibility of AHM was assessed twice by the same reader at an interval of 1 month. A κ value of 0.2 or less was defined as poor reliability, a κ value between 0.20 and 0.40 was defined as fair reliability, a κ value between 0.41 and 0.60 was defined as moderate reliability, a κ value between 0.61 and 0.80 was defined as good reliability, and a κ value of more than 0.8 was defined as excellent reliability [18].

### 2.3. Examinations

In eyes undergoing cataract surgery, the degree of cataract was assessed according to the Lens Opacities Classification System III (LOCS III) with slitlamp microscope [19]. Preoperatively, keratometry and axial length measurement were conducted. Surgical time, occurrence of intraoperative complications, and postoperative outcomes were recorded, such as aqueous flare intensity (four-point scale from 0 to 3) at 1 day postoperatively, intraocular pressure at 1 day and 1 month postoperatively, and the amount of mean relative and absolute refractive error (difference between target refraction and postoperative manifest refraction at 1 month after surgery).

### 2.4. Statistical Analysis

Numerical data are expressed as mean ± standard deviation. Statistical comparisons between two groups were performed using the Mann–Whitney U test. The categorical data were compared between groups with the χ^2^ test or Fisher’s exact test.

The logistic regression analysis using the backward-elimination method was performed to evaluate the influence of various factors on the visibility of AHM (dependent variable) in eyes that were undergoing cataract surgery. Explanatory variables included age of subjects; gender; axial length; corneal power; corneal cylinder; and LOCS III gradings for nuclear color, nuclear opalescence, cortical cataract, and posterior subcapsular cataract. Statistical analysis was conducted using SPSS Statistics for Windows software (version 27, IBM Corp., Armonk, NY, USA). A *p*-value of less than 0.05 was considered statistically significant.

A power calculation using a significance level of 5% (α) and a power of 95% (1-β) showed that a sample size of 73–126 would be required to estimate the population rate of AHM. In this calculation, AHM rate was assumed to be 4–9% based on our preliminary study.

## 3. Results

### 3.1. Demographics and Background Data

A total of 624 eyes of 624 patients were included. Among them, 223 eyes of 223 patients underwent cataract surgery by phacoemulsification and intraocular lens implantation. Patient’s demographics are shown in Table 1 and Table 2.

### 3.2. Intrarater Reliability

Intrarater reliability evaluated in 200 eyes at an interval of 1 month was excellent with the prevalence-adjusted, bias-adjusted kappa value of 0.90.

### 3.3. Visibility of Anterior Hyaloid Membrane

**Figure 1:**
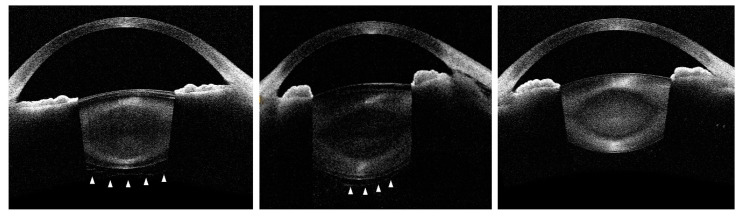
Representative AS-OCT images. (**Left**): Complete detachment of anterior hyaloid membrane (AHM) was observed (arrow heads). (**Middle**): AHM detachment was partially seen (arrow heads). (**Right**): AHM was not visible.

Figure 1 shows representative cases with and without visualization of AHM. Among the 624 eyes, AHM was seen in 43 eyes (6.9%). The patients with AHM detected (68.1 ± 8.8 years old) were significantly older than those without AHM detected (52.6 ± 25.6 years old) (*p* < 0.001). 

### 3.4. Analysis in Eyes Undergoing Cataract Surgery

The subgroup analysis in patients who underwent cataract surgery demonstrated that AHM was detected in 18 eyes (8.1%) in the preoperative AS-OCT imaging. Logistic regression analysis revealed that parameters significantly associated with AHM visibility were axial length (exp (B) = 1.315, *p* = 0.030) and corneal power (exp (B) = 1.480, *p* = 0.043). The eyes with visible AHM had greater axial length (24.9 ± 2.1 mm vs. 24.2 ± 1.9 mm) and higher corneal power (44.62 ± 1.41 D vs. 44.19 ± 1.47 D). Other parameters were not considered for the regression model.

Intraoperative and postoperative parameters are summarized according to the visibility of AHM in Table 3. The amount of mean absolute refractive error from target was significantly larger in eyes with visible AHM than in those with invisible AHM (*p* = 0.037), but the amount of mean relative refractive error from target was not significantly different between two groups (*p* = 0.602). Visibility of AHM did not influence other intraoperative and postoperative parameters, such as surgical time, occurrence of intraoperative complications, postoperative aqueous flare intensity, changes in intraocular pressure, and development of cystoid macular edema.

## 4. Discussion

We evaluated the visibility of AHM in the retrolental space of normal human eyes, finding that AHM was seen in 6.9% of eyes examined with the deep-range AS-OCT. While there have been a few case reports of spontaneous occurrence of AHM detachment and manifestation of Berger’s space in normal phakic eyes [13,20], no large-scale studies have been conducted on the prevalence of AHM detachment due to the difficulty of visualizing AHM in vivo. With the latest AS-OCT technology, we were able to non-invasively depict AHM that was detached from the posterior lens capsule. Although dehiscence of the Wieger ligaments from the lens capsule cannot be proven with the current AS-OCT, we suppose that observed AHM detachment herein is indicative of the presence of anterior vitreous detachment since obtained images are compatible with those reported in a previous histopathological study [7].

In the current study, AHM was more frequently seen in older patients and in eyes with greater axial length. These results are in good agreement with the reports of posterior vitreous detachment (PVD), in which PVD is more prevalent in older patients and in myopic eyes [21,22,23,24]. For PVD to occur, two different events must occur, liquefaction of the vitreous body (synchisis) and the weakening of adhesion between the vitreous posterior cortex and the internal limiting lamina [25,26,27]. Although little is known about AHM detachment from the posterior lens capsule, we assume that similar mechanisms play a role in the development of age-related AHM detachment. In our study, corneal power was also found to be relevant to AHM detection. At present, we have no clear explanation for this association, but it is assumed that higher corneal power is possibly related to the anteroposterior elongation of anterior ocular structures independent of axial length.

Using microscope-integrated intraoperative OCT during cataract surgery, Anisimova et al. [6]. demonstrated penetration of crystalline lens microfragments, cellular material, or medical suspension (triamcinolone) into the space between the posterior lens capsule and the anterior hyaloid membrane (Berger’s space), reporting that Wieger ligament rupture can allow excessive hydration of Berger’s space during phacoemulsification, leading to anterior displacement of the posterior lens capsule and thereby increasing the risk of instrument touch and posterior capsule rupture. In the current series, we experienced similar cases in which AHM detachment was indicated in the preoperative AS-OCT imaging and excessive undulation of the posterior capsule occurred during surgery. Since intraoperative complications could be avoided in those cases, there was no association between the presence of AHM detachment and incidence of intraoperative complications in our study. Nevertheless, preoperative evaluation of AHM detachment would be beneficial to forecast and brace for the risk factors of intraoperative complications. Further studies are awaited to elucidate these points.

In eyes that underwent cataract surgery, the amount of mean absolute refractive error from target at 1 month postoperatively was significantly larger in eyes with visible AHM than in those without visible AHM (*p* = 0.037), but the amount of mean relative refractive error from target was not significantly different between two groups (*p* = 0.602). These results indicate that AHM detachment is associated with lower accuracy of postoperative refraction, but no apparent tendency existed as to the myopic or hyperopic shift in those eyes. In eyes undergoing combined vitrectomy and cataract surgery, postoperative refraction is reported to be less accurate compared to cataract surgery alone, in which changes in the effective lens position after combined phacovitrectomy plays an important role [28,29]. In our study, it seems that the effective lens position was less predictive in eyes with visible AHM due to the lack of support for the posterior lens capsule by AHM attachment. This assumption, however, cannot be proven at present because we did not measure anterior chamber depth after cataract surgery in this study.

The retrolental space was imaged through the crystalline lens, and thus there is a possibility that presence of opaque lens might have influenced the visibility of AHM in our patients. In the subgroup analysis of pre-cataract surgery patients, however, detection rate of AHM was not influenced by the LOCS III gradings for nuclear color, nuclear opalescence, cortical cataract, and posterior subcapsular cataract. In addition, eyes with advanced cataract were precluded from the subjects. It is therefore unlikely that the degree of lens opacity significantly influenced the current results.

There are several limitations to this study. First, visibility of AHM and Berger’s space does not necessarily mean disruption of the ligament of Wieger. Direct visualization of the ligament of Wieger in vivo is highly difficult. A novel imaging modality or histopathological approach will be needed to assess the status of the ligament of Wieger. Second, being a retrospective study, the current study lacks several important data, such as postoperative anterior chamber depth, which is crucial to discuss the mechanisms underlying less accurate postoperative refraction in eyes with AHM detachment. A prospective study is required to clarify this point. Third, age of subjects was significantly associated with visibility of AHM in the analysis of the whole study population, but not in the subgroup analysis with pre-cataract surgery eyes. This may have been due to the smaller age range in patients undergoing cataract surgery (70.1 ± 10.9 years) than in the whole study population (53.7 ± 25.1 years), as well as smaller study size in the former group.

## 5. Conclusions

Retrolental imaging with the deep-range AS-OCT demonstrated that AHM and Berger’s space were visible in 6.9% of normal phakic eyes. The factors relevant to the visibility of AHM included age of patients and axial length. AHM detachment was associated with lower accuracy of postoperative refraction, but no apparent tendency existed as to the myopic or hyperopic shift in those eyes.

## Figures and Tables

**Table 1:** Demographic data of the whole study population.

Demographic Data	624 Eyes of 624 Patients
Age	53.7 ± 25.1 (5 to 96)
Male/female	301/323
DCVA (logMAR)	0.336 ± 0.386
Intraocular pressure (mmHg)	15.1 ± 3.1

Mean ± standard deviation (range), DCVA: distance-corrected visual acuity, logMAR: logarithm of minimum angle of resolution.

**Table 2:** Demographic data of eyes undergoing cataract surgery.

Demographic Data	223 Eyes of 223 Patients
Age	70.1 ± 10.9 years (25 to 93)
Male/female	96/127
DCVA (logMAR)	0.308 ± 0.335
Intraocular pressure (mmHg)	15.1 ± 2.8
Corneal power (diopter)	44.22 ± 1.47
Corneal cylinder (diopter)	0.94 ± 0.71
Axial length (mm)	24.34 ± 1.90
LOCS III grading	
Nuclear color (1~6)	1/62/124/20/12/4
Nuclear opalescence (1~6)	1/59/125/22/12/4
Cortical cataract (1~5)	79/33/41/67/3
Posterior subcapsular cataract (1~5)	149/31/17/16/10

Mean ± standard deviation (range), DCVA: distance-corrected visual acuity, logMAR: logarithm of minimum angle of resolution, LOCS III: Lens Opacities Classification System III.

**Table 3:** Intraoperative and postoperative parameters.

Parameters	AHM Visible (18 Eyes)	AHM Invisible (205 Eyes)	*p*-Value
Surgical time (minute)	14.9 ± 10.0	15.5 ± 15.5	0.540
Posterior capsule rupture	0%	1.5%	0.776
Cystoid macular edema	0%	0.5%	0.919
Aqueous flare intensity at 1 day postoperatively (0~3)	0/14/4/0	0/158/46/1	0.956
Changes in intraocular pressure at 1 day postoperatively (mmHg) *	5.6 ± 6.3	3.9 ± 5.0	0.147
Changes in intraocular pressure at 1 month postoperatively (mmHg) *	−1.6 ± 2.5	−1.7 ± 2.7	0.634
Relative refractive error at 1 month postoperatively (diopter)	−0.048 ± 0.788	0.099 ± 0.636	0.602
Absolute refractive error at 1 month postoperatively (diopter)	0.670 ± 0.384	0.494 ± 0.412	0.037

AHM: anterior hyaloid membrane. * Comparison with preoperative intraocular pressure.

## Data Availability

The datasets generated during and/or analyzed during the current study are available from the corresponding author on reasonable request.
